# Resistance on the Latest Oral and Intravenous P2Y12 ADP Receptor Blockers in Patients with Acute Coronary Syndromes: Fact or Myth?

**DOI:** 10.3390/jcm11237211

**Published:** 2022-12-04

**Authors:** Peter Blaško, Matej Samoš, Tomáš Bolek, Lucia Stančiaková, Ingrid Škorňová, Martin Jozef Péč, Jakub Jurica, Ján Staško, Marián Mokáň

**Affiliations:** 1 Department of Internal Medicine I, Jessenius Faculty of Medicine in Martin, Comenius University in Bratislava, 036 59 Martin, Slovakia; 2 Out-Patient Clinic of Cardiology, 957 01 Banovce nad Bebravou, Slovakia; 3 Department of Hematology and Blood Transfusion, National Centre of Hemostasis and Thrombosis, Jessenius Faculty of Medicine in Martin, Comenius University in Bratislava, 036 59 Martin, Slovakia

**Keywords:** prasugrel, ticagrelor, cangrelor, high on-treatment platelet reactivity, stent thrombosis, acute coronary syndrome

## Abstract

Novel P2Y12 ADP receptor blockers (ADPRB) should be preferred in dual-antiplatelet therapy in patients with acute coronary syndrome. Nevertheless, there are still patients who do not respond optimally to novel ADP receptor blocker therapy, and this nonoptimal response (so-called “high on-treatment platelet reactivity” or “resistance”) could be connected with increased risk of adverse ischemic events, such as myocardial re-infarction, target lesion failure and stent thrombosis. In addition, several risk factors have been proposed as factors associated with the phenomenon of inadequate response on novel ADPRB. These include obesity, multivessel coronary artery disease, high pre-treatment platelet reactivity and impaired metabolic status for prasugrel, as well as elderly, concomitant therapy with beta-blockers, morphine and platelet count for ticagrelor. There is no literature report describing nonoptimal therapeutic response on cangrelor, and cangrelor therapy seems to be a possible approach for overcoming HTPR on prasugrel and ticagrelor. However, the optimal therapeutic management of “resistance” on novel ADPRB is not clear and this issue requires further research. This narrative review article discusses the phenomenon of high on-treatment platelet reactivity on novel ADPRB, its importance in clinical practice and approaches for its therapeutic overcoming.

## 1. Introduction

Novel P2Y12 ADP receptor blockers (ADPRB), namely prasugrel, ticagrelor and cangrelor, have emerged as a potent therapeutic approach for ADP signaling pathway inhibition in patients with acute coronary syndromes (ACSs), with [1,2,3] or without planned percutaneous coronary intervention (PCI) [3], or patients who undergo PCI without oral ADPRB pre-treatment [4]. This therapy should be considered, especially in those patients who do not respond optimally to clopidogrel (patients with clopidogrel high on-treatment platelet reactivity = “clopidogrel resistance”) [5]. Nevertheless, there are still patients who do not respond optimally to novel ADPRB therapy, and this nonoptimal response could be connected with increased risk of adverse ischemic events, such as myocardial re-infarction, target lesion failure and stent thrombosis [6,7]. This article discusses the phenomenon of “resistance” (high on-treatment platelet reactivity) on novel-generation ADPRB (the latest ADPRB available in clinical practice), its importance in clinical practice and approaches for its therapeutic overcoming.

## 2. Methods

The aim of this article is to provide a brief traditional (narrative) review, which summarizes current data regarding the prevalence and clinical significance of HTPR on novel-generation ADPRB, namely prasugrel, ticagrelor and cangrelor, in patients with acute coronary syndrome. To achieve this aim, the most relevant medical scientific literature databases—Web of Science, PubMed and Scopus—were searched, using selected keywords: “high on treatment platelet reactivity” or “resistance” or “insufficient response” and “prasugrel” or “ticagrelor” or “cangrelor” and “acute coronary syndrome” or “myocardial infarction” or “STEMI” or “NSTEMI” or “unstable angina”. If needed, additional keywords, such as “major adverse cardiac event” or “stent thrombosis” or “stent failure” or “target lesion failure” were added, and the literature was researched. The authors non-systematically identified relevant articles matching their aim. Subsequently, a review of findings from these articles was provided, together with a discussion of the clinical implications of published observations.

## 3. Insufficient Response to ADPRB and the Risk of Future Events in Patients with ACS

ADPRB treatment failure is a major risk factor for stent thrombosis and early PCI failure [8]. Patients with high on-treatment platelet reactivity (HTPR) have an approximately 2–3-fold higher risk of adverse ischemic events and stent thrombosis than those without HTPR. Moreover, ADPRB HTPR has been observed in patients with ACS previously independently associated with unfavorable in-hospital clinical outcome [9], with increased risk of long-term thrombotic events in patients with implanted drug-eluting stents [10,11], connected with frequent recurrent angina and left ventricular failure [12] and predicted future cardiovascular events after PCI for ACS [13]. A previous observational study [14] showed that clopidogrel resistance was present in 72.5% of patients admitted for repeated ACS, which suggests that HTPR likely plays an important role in recurrent ACS. Additionally, the phenomenon was reported as a leading cause of stent thrombosis (including left main-chain and multi-vessel stent thrombosis) in several clinical cases [15,16,17,18,19].

Despite the abovementioned evidence, there is still an ongoing discussion about the association between antiplatelet drugs HTPR and major adverse cardiovascular events (MACE), and the clinical implication of antiplatelet therapy HTPR is not fully determined. Several factors could be responsible for this ambiguity. First, the results of so-far published studies are controversial (especially for aspirin HTPR), and there are studies that did not confirm the relation between HTPR and the risk of future ischemic adverse events [20,21,22,23]. Second, a MACE in a patient after previous coronary intervention is a complex phenomenon, which could be associated with stent failure itself (due to either stent thrombosis or stent restenosis) or due to “de novo” atherothrombotic events, which could develop due to progression of plaque instability or due to platelet activation or both mechanisms can be involved. Additionally, stent thrombosis, for example, could be connected with HTPR, stent malposition, inadequate stent expansion, stent undersizing, small stent diameter, stent fracture, edge dissection or drug non-compliance [24,25]. Therefore, one needs to understand that HTPR on ADPRB therapy is just one risk factor in a complex clinical problem. Third, there is still no definite answer for how to deal with HTPR, as multiple previously tested approaches failed to improve clinical outcomes [26,27,28]. Considering the fact that failure to reduce platelet reactivity in the settings of HTPR seems to be an independent predictor of future MACE [29], the issue of not having an optimal algorithm for HTPR-guided intensification of platelet inhibition could definitely play an important role in these uncertainties.

Fourth, there is an issue in laboratory testing for HTPR detection. In fact, various platelet function tests (PFTs) with different test principles have been tested (and validated) for the detection of HTPR [30]. Some of them are designed as point-of-care tests, others require complex laboratory equipment and skilled staff to perform the examination. Light transmission aggregometry (LTA) with a specific inducer (adenosine diphosphate—ADP) is still recognized as a standard laboratory test for PFT, which also includes the detection of HTPR, while the Vasodilator-Stimulated Phosphoprotein phosphorylation (VASP-P) by flow cytometric analysis is probably the most specific test for assessing the rate of P2Y12 ADP signaling pathway inhibition [31]. Both tests have important limitations (especially in the settings of daily clinical practice, including the need for measuring the antiplatelet drug response in a 24/7 ACS program), such as the need for special equipment, skilled staff, time demand and, in the case of LTA, the need to process the sample immediately (within first hour from blood sampling). Therefore, several point-of-care assays, namely Multiplate^®^, VerifyNow^®^, PFA-100^®^ and platelet mapping thromboelastography^®^ (TEG^®^), have been designed. Multiplate^®^ (Roche Diagnostics, Indianapolis, IN, USA) is a point-of-care assay, which tests citrated whole blood samples using the electrical impedance aggregometry principle. The assay uses platelet stimulation with specific inducers (ADP) to activate platelet aggregation. Once the aggregated platelets attach the sensor wires in the Multiplate^®^ device, electrical resistance (impedance) is detected and displayed as aggregation units (AUs) against time (area under the curve = AUC) [32]. VerifyNow^®^ (Werfen, Barcelona, Spain) assay is performed as a point-of-care test using a citrated whole blood sample. In this turbidimetry-based assay, ADP induction is used to initiate platelet aggregation on fibrinogen-coated beads. Platelet aggregation is determined by the percentage of the light transmission and expressed in P2Y12 reaction units (PRUs). Low PRU indicates the high P2Y12 receptor inhibition and better response to P2Y12 ADPRB. VerifyNow^®^ assay is a rapid test, which can be performed even at the bedside within 5 min, which is an advantage when compared with LTA and VASP-P assays. Moreover, the examination itself (due to a simple technique) and the interpretation of results can be carried out easily (there is no need for skilled staff to perform and/or evaluate the results). Both assays have been used for PFT in post-marketing studies, including randomized ones [26,30,32]. Platelet function assay-100 = PFA-100^®^ (Siemens Medical Solutions, Malvern, PA, USA) is another point-of-care assay, which can be used to monitor the effect of P2Y12 ADPRB. The assay uses citrated whole blood sample and measures the platelet aggregation and effect of antiplatelet agents under higher shear stress. This test can be performed rapidly (in less time) and, similarly to VerifyNow^®^ assay, has a simple test technique, which is an added advantage when compared with conventional PFT. PFA-100^®^ has collagen-coated, epinephrine-coated and ADP-coated cartridges. If ADPRB is present in a sample, the blood will flow under higher shear rate through the capillary and through a small aperture of the PFA-100^®^ analyzer. Subsequently, platelets will aggregate and form the ADP-induced platelet plug by blocking the aperture. The time taken for complete occlusion of the aperture is recorded as closure time (CT). Prolonged CT indicates a better response to ADPRB [32]. PFA-100^®^ assay has been used in clinical studies mostly for the detection of aspirin resistance [33,34]; however, there are data to show that the test can be used also for the determination of response to ADPRB [35]. On the other side, it is questionable whether the use of point-of-care assays (compared to traditional PFT) has an impact (negative) on the clinical utility of PFT studies, as these assays are criticized for their limited sensitivity and/or specificity [36,37]. Additionally, it is possible that a single PFT will not reliably detect HTPR, and that confirmation of suspected HTPR with another PFT might be needed for establishment of final diagnosis (confirmation of results obtained from a point-of-care test by a laboratory-based test). Finally, there is an issue of inconsistent cut-off values for the detection of HTPR, especially when point-of-care assays are used for its determination. For example, for the VerifyNow^®^ assay, cut-off values of >280 PRU, >272 PRU, >235 PRU and >230 PRU have been used to define HTPR in previously published studies [26,30]; for Multiplate^®^, at least two cut-off values (468 AUC, 450 AUC) have been reported. All these unclosed issues could explain the ongoing discussion regarding the clinical implication of HTPR.

Nonetheless, considering the fact that, with clopidogrel, ADP-induced platelet aggregation remains significantly high in ACS patients, even after 48 h from standard loading [38], it is unsurprising that a novel generation of ADPRB (Table 1) with more rapid and more potent platelet inhibitory effects has been developed and introduced to clinical practice, especially for the treatment of patients with ACS.

## 4. Novel-Generation ADPRB

### 4.1. Prasugrel

Prasugrel is an irreversible, 3^rd^-generation thienopyridine P2Y12 ADPRB, which is indicated for combined (with aspirin) antiplatelet therapy in PCI-treated patients with ACS [1,2,39]. Prasugrel [39] provides more consistent inhibition of the P2Y12 ADP receptor and has lower intraindividual variability in efficacy compared with clopidogrel. It is hydrolyzed by plasma esterases, then metabolized by cytochrome P450 (CYP) 3A4 and 2B6 enzymes to form an active metabolite and has a plasma half-life of 7.4 h. The inactivation of the active metabolite is mediated trough drug S-methylation and drug conjugation. The inactive metabolites are excreted by urine (68%) and stool. The response to prasugrel is not affected by CYP 2C19 inhibition, loss of CYP 2C19 gene function or decreased function of P-glycoprotein (P-gp). Loading doses of 60 mg of prasugrel reach, in theory, full antiplatelet effects 15–30 min after administration. The benefit of prasugrel therapy seems to be the highest in patients with diabetes mellitus [1]. Prasugrel therapy was repeatedly used to overcome clopidogrel resistance [15,19].

### 4.2. Ticagrelor

Ticagrelor is a reversible, 3^rd^-generation non-thienopyridine P2Y12 ADPRB, approved for combined antiplatelet therapy in patients with ACS (with or without PCI) and for long-term combined antiplatelet therapy in coronary artery disease (CAD) patients with high ischemic and low bleeding risk [3,40]. Ticagrelor offers rapid and consistent inhibition of the P2Y12 ADP signaling pathway, which is independent of previous metabolic activation and P-gp function. Ticagrelor reaches its maximal plasma activity approximately 1.5 h after ingestion and has a plasma half-life of 8.5 h. However, it undergoes metabolism, which is mediated by CYP 3A4 and, therefore, strong inducers/inhibitors of this enzyme complex could affect the concentrations of ticagrelor and lead to unexpected drug activity. Ticagrelor is, after metabolic transformation, eliminated by hepatic and renal excretion. A loading dose of 180 mg followed by a maintenance dose of 90 mg twice daily are recommended for patients with ACS, while a dose of 60 mg twice daily is recommended for long-term antiplatelet prophylaxis in high-ischemic-risk CAD patients [5,39,40]. Although a recent randomized study suggested better efficacy (with similar safety profile) of prasugrel compared to ticagrelor in ACS patients undergoing PCI [2], ticagrelor therapy had been repeatedly described as an effective approach for overcoming clopidogrel resistance [5,41,42] and is still preferred in those ACS patients who could not receive prasugrel.

### 4.3. Cangrelor

Cangrelor is a reversible, parenteral, 3rd-generation non-thienopyridine P2Y12 ADPRB, which is indicated for combined antiplatelet therapy in patients undergoing PCI (both for ACS and stable CAD) who are not pre-treated with oral ADPRB [4]. Cangrelor reaches the maximal antiplatelet effect within 2 min after bolus injection (30 ug per kg of body weight) and has a very short plasma half-life (2.6–3.3 min) requiring continuous intravenous infusion (4 ug per kg of body weight per minute) to maintain adequate ADP receptor inhibition. Cangrelor does not require metabolic transformation to form an active metabolite and is independent of CYP and P-gp activity. Cangrelor is deactivated by plasmatic de-phosphorylation; the inactive metabolite is eliminated by urine (58%) and stool (35%). Normal platelet function is restored within 1 h after stopping the cangrelor infusion [43]. Cangrelor administration could be, in theory, used as a bailout option for overcoming clopidogrel resistance in patients presenting with stent thrombosis [44].

## 5. Prasugrel Resistance in Patients with ACS

In one of the first studies examining the prevalence of insufficient response on prasugrel, Bonello et al. [45] reported that a significant portion of patients undergoing PCI for ACS did not achieve optimal platelet inhibition. In this study, with 301 patients receiving a prasugrel loading dose of 60 mg, 25.2% of patients had HTPR. Patients who experienced a thrombotic event after PCI had significantly higher residual platelet activity measured with VASP-P compared with those free of adverse thrombotic events. In our previous prospective study, which aimed to map the platelet reactivity in novel ADP receptor-blocker-treated patients with acute ST elevation myocardial infarction (STEMI), 60.9% of prasugrel-treated patients did not achieve sufficient platelet inhibition after a loading dose of 60 mg (measured 1.6 ± 0.7 h after loading dose administration) and 8.7% of prasugrel-treated patients remained non-responders in second blood sampling performed after 20.4 ± 2.6 h from loading dose administration [46]. In addition, Aradi et al. [47] tested platelet reactivity with LTA and whole blood impedance aggregometry (Multiplate^®^) in 103 consecutive, high-risk ACS patients 12 to 24 h after administration of loading dose (60 mg) of prasugrel. The authors of this study reported significant inter-patient variability in platelet reactivity after all doses of prasugrel, and the prevalence of HTPR was significantly higher during the maintenance dose administration. On the other side, another study enrolling PCI-treated ACS patients receiving prasugrel reported HTPR (defined as VASP-P index > 50%) 2 to 4 weeks after hospital discharge only in 6.8% of patients [48]. Similarly, only 9.1% of acute STEMI patients was identified as a non-responders on prasugrel (defined as VASP-P index > 50% 6 to 12 h after prasugrel loading dose administration) in a previous prospective study performed by Laine et al. [49]. A previous meta-analysis of 14 studies with 1822 patients reported the HTPR in 9.8% of prasugrel-treated patients [50]; Siller-Matula et al. [51] reported that only 3% of prasugrel-treated patients had HTPR in the maintenance phase of treatment. In another single-center retrospective analysis of 809 PCI-treated ACS patients, the prevalence of prasugrel HTPR was even lower and only 1.6% of prasugrel-treated patients fulfilled the criteria for HTPR [52]. However, this study measured platelet reactivity with whole blood impedance aggregometry. In a more recent analysis, Verdoia et al. [53,54], in their prospective studies, reported that HTPR on prasugrel was observed in 10% and 12.3% of patients, respectively. In these studies, whole blood impedance aggregometry was used for determination of HTPR.

Based on these data (Table 2), one could conclude that, although with significantly lower prevalence (compared to clopidogrel), HTPR on prasugrel exists, and its prevalence varies from 1.6 to approximately 25% of treated patients (depending on platelet function test used and chosen time from loading dose to blood sampling). In addition, if the time interval from drug administration to blood sampling is too short, the number of patients with inadequate response can be even higher [46]. Nevertheless, it seems that the phenomenon of prasugrel HTPR is connected with adverse ischemic events. This observation was repeatedly reported in previous prospective studies, including sub-analyses of randomized studies [45,55,56]. Aradi et al. [56], for example, reported, in a pre-specified exploratory analysis of the TROPICAL-ACS trial, that, although infrequent, prasugrel HTPR was connected with increased risk of thrombotic events. The association between prasugrel HTPR and the higher risk of adverse ischemic events post PCI is also supported by multiple reported clinical cases of prasugrel “resistance”, which consistently reported serious adverse thrombotic events, including repeated cases of stent thrombosis [6,57,58]. Hence, the HTPR on prasugrel could probably be considered as a risk factor for adverse ischemia/thrombosis, similarly to clopidogrel HTPR.

**Figure 1:**
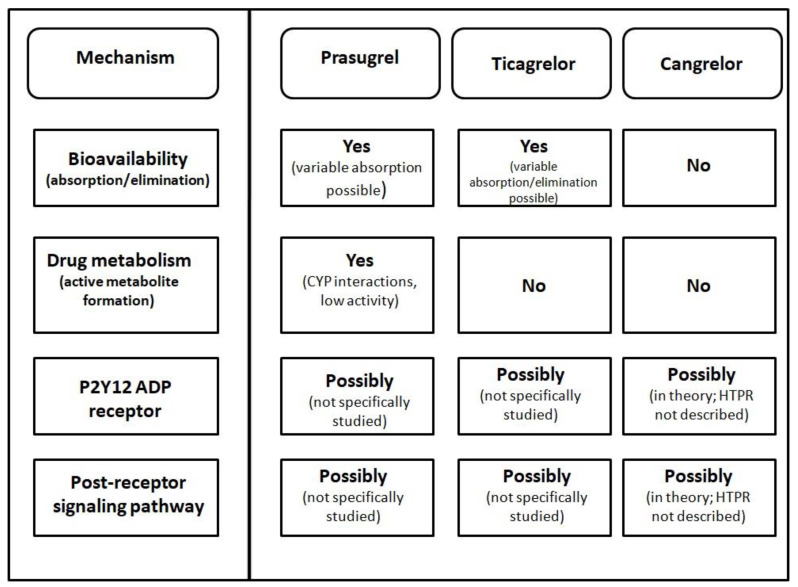
Possible mechanism of HTPR on novel-generation ADPRB [59]. ADP—adenosine diphosphate; ADPRB—P2Y12 ADP receptor blockers; CYP—cytochrome P450 enzyme; HTPR—high on-treatment platelet reactivity.

A question regarding the mechanism of prasugrel HTPR could be posed. In fact, right now, no satisfactory answer to this question exists, as there is no study examining this problem. Theoretically, several factors could be responsible for this insufficient drug response (Figure 1). Prasugrel HTPR can be caused by decreased bioavailability—either due to decreased absorption or increased drug elimination, by impaired drug metabolism (either due to genetic polymorphism or drug interactions), leading to decreased formation of an active metabolite, due to ineffective inhibition of the platelet P2Y12 ADP receptor via an active metabolite or due to impaired response on ADP receptor inhibition on the level of the post-receptor signaling pathway [59].

## 6. Ticagrelor Resistance in Patients with ACS

Although first observations reported no risk of HTPR on ticagrelor [60,61], Laine et al. [62] subsequently reported, in their multicenter prospective observational study enrolling 115 ticagrelor-treated patients, that platelet inhibition post 180 mg of ticagrelor loading dose is not uniform and that 3.5% of patients had HTPR (defined as VASP-P index > 50%, blood sampling was performed 6 to 24 h post drug loading). In our previously mentioned analysis of STEMI patients planned for primary PCI [46], platelet inhibition after 180 mg of ticagrelor loading dose was not sufficient in 42.9% of patients in a sample taken 1.4 ± 0.6 h and in 14.3% of patients in a sample taken 21.0 ± 2.0 h post drug loading, respectively. In other studies, the range of ticagrelor HTPR after loading dose administration ranged from 1.5 to 60.2%, depending on studied patient population, method used for HTPR detection and timing of blood sampling [50,51,63,64,65,66,67]. Although Verdoia et al. reported in their studies [54,68,69] 8.6 to 13.7% prevalence of ticagrelor HTPR on maintenance dosing (tested 30 to 90 days post drug loading), it seems that a longer duration of ticagrelor therapy probably achieves sufficient platelet inhibition in the majority of patients, as the majority of so-far published studies, including meta-analyses, reported very low rates (0.0–1.9%) of ticagrelor HTPR (Table 3) on long-term therapy [49,52,70,71,72,73,74,75,76]. This prevalence practically limits the occurrence of ticagrelor HTPR to occasional clinical cases. However, although the evidence is still limited and the phenomenon of ticagrelor resistance is relatively rare, ticagrelor HTPR seems to be connected with a higher risk of adverse ischemic events [67], very similarly to clopidogrel HTPR and prasugrel HTPR. Additionally, Musallam et al. reported a case of a patient with subacute stent thrombosis in whom ticagrelor HTPR was verified [7], and Malik [77] described a case of stent thrombosis 22 days after successful drug eluting coronary stent implantation in a 62-year-old man with diabetes who did not respond adequately to ticagrelor therapy. In this particular case, intravascular imaging with optical coherence tomography was performed, and stent underexpansion, stent strunt mal-apposition and edge dissection were excluded. Finally, Jariwala et al. [78] described a case of subacute stent thrombosis after uncomplicated implantation of sirolimus-eluting coronary stent, which developed despite ticagrelor therapy (in this case, authors were unable to verify HTPR with laboratory testing). In summary, current evidence suggests that ticagrelor HTPR exists, but its prevalence is lower compared to clopidogrel and prasugrel HTPR. Nevertheless, this phenomenon seems to be connected with a higher risk of ischemic adverse events, including stent thrombosis.

Looking at the mechanism of ticagrelor HTPR, it is obvious that impaired (pro)drug conversion (metabolism) does not play a role, as ticagrelor is an active metabolite, which does not require metabolic transformation to achieve its drug activity. In theory, all the other possible mechanisms discussed in connection with prasugrel HTPR (Figure 1) could be responsible [59]. Nevertheless, similarly to prasugrel HTPR, there is no study specifically examining the mechanism of this HTPR; therefore, clarification of this matter is still open for future research.

## 7. Cangrelor Resistance in Patients with ACS

Looking at the currently available data, there is no study examining the prevalence of cangrelor HTPR in patients with acute coronary syndrome, no study describing this phenomenon or clinical case report describing a case of ischemic adverse post-PCI event related to failure of cangrelor therapy. Moreover, cangrelor seems to be a promising agent for treatment (overcoming) prasugrel HTPR [79,80] or to bridge the gap until optimal platelet inhibition with ticagrelor is achieved [81]. Nevertheless, cangrelor has been approved for clinical use in patients undergoing PCI relatively recently; thus, it might be possible that the phenomenon of cangrelor HTPR just awaits its description.

## 8. Risk Factors of Novel ADPRB Resistance

The next question should be what are the risk factors for HTPR on novel-generation ADPRB? Searching the literature, several factors have been proposed as factors associated with this phenomenon. For prasugrel, obesity (BMI > 30 kg/m^2^) [48,82,83,84], multivessel coronary artery disease [48], carrying a CYP 2C19*2 or 2C19*17 loss-of-function allele [85,86], high pre-treatment platelet reactivity and smoking status [87] and impaired metabolic status (with higher levels of glycosylated hemoglobin and low-density lipoprotein cholesterol) [53] have been reported as factors independently predicting HTPR. Several other clinical factors, namely chronic kidney disease [53], type 2 diabetes [88], elderly and proton pump inhibition co-therapy [47], were studied, but an association was not found. For ticagrelor, in a previous study, age and BMI positively and smoking negatively affected on-treatment platelet reactivity [71]. Verdoia et al. [68] reported that, using multivariable analysis, age (≥70 years), concomitant therapy with beta-blockers and platelet count independently predicted HRPR on ticagrelor. In another prospective study in ticagrelor-treated ACS patients, Adamski et al. reported that the presence of ST-segment elevation and morphine co-administration were the strongest predictors of ticagrelor HTPR [89]. Other clinical factors, such as chronic kidney disease, diabetes or proton pump inhibitor co-administration, probably do not affect the efficacy of ticagrelor therapy [88,90,91].

## 9. How to Manage Insufficient Response to Novel ADPRB?

In theory, novel-generation ADPRB HTPR can be managed by modification of drug dosing (either with re-loading or increasing the maintenance dosage) [92], by adding other antithrombotic agents, to bridge the time until the drug achieves its full activity (glycoprotein IIb/IIIa inhibitor or parenteral ADPRB—cangrelor) [93] or to achieve more potent platelet inhibition in long-term therapy (cilostazol or low-dose rivaroxaban) [94], or by switching the novel-generation ADPRB (prasugrel to ticagrelor in prasugrel HTPR and ticagrelor to prasugrel in ticagrelor HTPR) [54,95]. One must say that each of the strategies has its disadvantages and that none of them was tested in a randomized trial. For example, adding the third antithrombotic agent to long-term therapy can increase the risk of bleeding, while the effect on a reduction in adverse thrombotic events remains unclear. In addition, increasing the drug dose leads to long-term drug dosing, for which efficacy and safety were not previously tested in clinical trials. Furthermore, cangrelor has been approved only in ADPRB-naïve patients who are planned for PCI, and the safety of its administration in those who already received ADPRB loading (although with insufficient platelet response) remains unclear. In the majority of so-far published cases [6,7,57,58,77,78], the authors used a switch strategy, either alone or with bridging the ineffective antiplatelet response with adding glycoprotein IIb/IIIa inhibition. This strategy appears to be safe and effective, as there were no reports of repeated ischemic or serious bleeding adverse events in these cases. However, the evidence for any approach is still limited to a small number of clinical cases, and further research on the issue of optimal management of HTPR on novel-generation ADPRB is definitely needed.

## 10. Conclusions

Based on the limited evidence discussed in this review article, we can conclude that the phenomenon of HTPR or resistance on novel-generation ADPRB therapy might exist. The prevalence of novel-generation ADPRB HTPR is lower compared to clopidogrel HTPR. Additionally, several studies suggested that this phenomenon could be connected with a high risk of adverse ischemic events; however, the evidence for this association is still limited. Therefore, there is a need for future research dedicated to clinical impact and optimal management of this phenomenon.

## Figures and Tables

**Table 1:** Novel-generation ADP receptor blockers in current clinical practice.

Drug	Route of Administration Dosing	Bioavailability	Receptor Inhibition	Time to Peak Platelet Inhibition	Clinical Application	HTPR (Prevalence)
Prasugrel	Oral Loading dose of 60 mg followed by 10 mg once daily (5 mg in elderly and low body weight)	Prodrug	Irreversible	0.5–2 h	ACS with PCI	Described (1.6–25%; higher if time from drug administration to blood sampling is too short)
Ticagrelor	Oral Loading dose of 180 mg followed by 90 mg twice daily (60 mg twice daily in CAD)	Direct-acting	Reversible	1.5–2 h	ACS High ischemic risk CAD	Described (8.6–13.7% if tested 30 to 90 days post drug loading; 0.0–1.9% on long-term therapy)
Cangrelor	Intravenous Bolus injection of 30 ug/kg followed by continuous intravenous infusion of 4 ug/kg/min.	Direct-acting	Reversible	2 min	PCI (if not pretreated with oral agent)	Not described

ACS—acute coronary syndromes; CAD—coronary artery disease; HTPR—high on-treatment platelet reactivity; PCI—percutaneous coronary intervention.

**Table 2:** Summary of studies reporting prasugrel HTPR.

Study	Type of Study	Studied Population	Number of Patients	Test for HTPR	Cut Off	Main Results
Bonello et al. [45]	Prospective multicenter (non-randomized)	PCI for ACS	301	VASP-P	VASP-P: PRI > 50%	HTPR in 25.2% of patients; significantly higher PRI in those with thrombotic events
Škorňová et al. [46]	Prospective single-center (non-randomized)	STEMI with primary PCI	44	LTA with ADP induction, VASP-P	LTA: >50%, VASP-P: PRI > 50%	HTPR in 8.7% of patients
Aradi et al. [47]	Prospective multicenter (non-randomized)	high risk ACS	104	LTA, Multiplate^®^	LTA: >46%, Multiplate^®^: >47 AU	inter-patient variability after prasugrel loading dosing; no effect of PPI on prasugrel activity
Cayla et al. [48]	Prospective two high-volume centers (non-randomized)	ACS with PCI	444	VASP-P, VerifyNow^®^, LTA	VASP-P: PRI ≥ 50%, VerifyNow^®^: ≥235 PRU, LTA: ≥46.2%	HTPR in 3.2–6.8% of patients according to method used for detection
Laine et al. [49]	Prospective single-center (randomized)	STEMI with primary PCI	44	VASP-P	VASP-P: PRI ≥ 50%	HTPR in 9.1% of patients
Lemesle et al. [50]	Meta-analysis (14 studies included)	CAD	1822	VASP-P, VerifyNow^®^	VASP-P: PRI ≥ 50%, different cut off for VerifyNow in included studies (208–235 PRU)	HTPR in 9.8% of patients
Siller-Matula et al. [51]	Prospective single-center (non-randomized)	ACS	200	Multiplate^®^	Multiplate^®^: >46 AU	HTPR in 3% of patients
Selhorst et al. [52]	Retrospective single-center	ACS with primary PCI	809	Multiplate^®^	Multiplate^®^: >468 AUC	HTPR in 1.6% of patients
Verdoia et al. [53]	Prospective single-center (non-randomized)	ACS with PCI	190	Multiplate^®^	Multiplate^®^: >417 AUC	HTPR in 10% of patients
Verdoia et al. [54]	Prospective single-center (non-randomized)	ACS with PCI	105	Multiplate^®^	Multiplate^®^: >417 AUC	HTPR in 12.3% of patients

ACS—acute coronary syndrome; ADP—adenosine diphosphate; AU—aggregation units; AUC—area under the curve; CAD—coronary artery disease; HTPR—high on treatment platelet reactivity; LTA—light transmission aggregometry; PCI—percutaneous coronary intervention; PRI—platelet reactivity index; PRU—P2Y12 reactivity units; VASP-P—Vasodilator-Stimulated Phosphoprotein phosphorylation.

**Table 3:** Summary of studies reporting ticagrelor HTPR.

Study	Type of Study	Studied Population	Number of Patients	Test for HTPR	Cut Off	Main Results
Alexopoulos et al. [60]	Prospective single-center (randomized)	ACS with PCI and HTPR on clopidogrel	44	VerifyNow^®^	VerifyNow^®^: ≥235 PRU	HTPR in 0% of patients
Alexopoulos et al. [61]	Prospective single-center (randomized)	ACS with PCI and T2D	30	VerifyNow^®^	VerifyNow^®^: ≥230 PRU	HTPR in 0% of patients
Laine et al. [62]	Prospective multicenter (non-randomized)	ACS with PCI	115	VASP-P	VASP-P: PRI ≥ 50%	HTPR in 3.5% of patients
Škorňová et al. [46]	Prospective single-center (non-randomized)	STEMI with primary PCI	44	LTA with ADP induction, VASP-P	LTA: >50%, VASP-P: PRI > 50%	HTPR in 14.3% of patients
Lemesle et al. [50]	Meta-analysis (14 studies included)	CAD	1822	VASP-P, VerifyNow^®^	VASP-P: PRI ≥ 50%, different cut off for VerifyNow in included studies (208–235 PRU)	HTPR in 1.5% of patients
Siller-Matula et al. [51]	Prospective single-center (non-randomized)	ACS	200	Multiplate^®^	Multiplate^®^: >46 AU	HTPR in 2% of patients
Laine et al. [63]	Prospective single-center (randomized)	ACS with PCI and T2D	100	VASP-P	VASP-P: PRI ≥ 50%	HTPR in 6% of patients
Verdoia et al. [64]	Prospective single-center (non-randomized)	ACS	190	Multiplate^®^	Multiplate^®^: >417 AUC	HTPR in 11% of patients
Barbieri et al. [65]	Prospective single-center (non-randomized)	PCI	537	Multiplate^®^	Multiplate^®^: >417 AUC	HTPR in 12.7% of patients
Li et al. [66]	Prospective single-center (non-randomized)	ACS	176	TEG^®^	TEG^®^: MA > 47 mm	HTPR in 3.98% of patients
Laine et al. [67]	Prospective multicenter (non-randomized)	ACS with PCI	530	VASP-P	VASP-P: PRI ≥ 50%	HTPR in 5.3% of patients
Verdoia et al. [54]	Prospective single-center (non-randomized)	ACS with PCI	105	Multiplate^®^	Multiplate^®^: >417 AUC	HTPR in 8.6% of patients
Verdoia et al. [68]	Prospective single-center (non-randomized)	ACS with PCI	195	Multiplate^®^	Multiplate^®^: >417 AUC	HTPR in 13.3% of patients
Verdoia et al. [69]	Prospective single-center (non-randomized)	ACS with PCI	432	Multiplate^®^	Multiplate^®^: >417 AUC	HTPR in 11.4% of patients
Laine et al. [49]	Prospective single-center (randomized)	STEMI with primary PCI	44	VASP-P	VASP-P: PRI ≥ 50%	HTPR in 0% of patients
Selhorst et al. [52]	Retrospective single-center	ACS with primary PCI	809	Multiplate^®^	Multiplate^®^: >468 AUC	HTPR in 1.9% of patients
Alexopoulos et al. [70]	Prospective single-center (non-randomized)	ACS with PCI	512	VerifyNow^®^	VerifyNow^®^: >208 PRU	HTPR in 0% of patients
Alexopoulos et al. [71]	Meta-analysis (8 studies included)	CAD or ACS (with or without PCI)	445	VerifyNow^®^	VerifyNow^®^: >230 PRU	HTPR in 0% of patients
Gaglia et al. [72]	Prospective single-center (non-randomized)	ACS and black rase	29	LTA, VASP-P, VerifyNow^®^	LTA: >60%, VASP-P: PRI > 50%, VerifyNow^®^: >208 PRU	HTPR in 0% of patients
Sweeny et al. [73]	post-hoc analysis of prospective multicenter study (randomized)	ACS (troponin negative) with PCI	100	VerifyNow^®^	VerifyNow^®^: >208 PRU	HTPR in 5.9% of patients
Liu et al. [74]	Prospective multicenter (randomized)	NSTE ACS	278	VASP-P	VASP-P: PRI ≥ 50%	HTPR in 3.1–5.3% of patients (according to ticagrelor loading dose)
Wen et al. [75]	Meta-analysis (14 studies included)	ACS	2629	VASP-P, VerifyNow^®^	VASP-P: PRI ≥ 50%, VerifyNow^®^: ≥208 PRU or ≥230 PRU	HTPR in 0.66–2.67% of patients (according to method used for detection)
Dai et al. [76]	Meta-analysis (25 studies included)	ACS	5098	VASP-P, VerifyNow^®^, Multiplate^®^	Not reported	low incidence of HTPR on ticagrelor maintenance dosing (exact rate was not reported)
Musallam et al. [7]	Case report	stent thrombosis on ticagleror therapy	1	VerifyNow^®^	-	HTPR (339 PRU) at the time of stent thrombosis
Malik [77]	Case report	stent thrombosis on ticagleror therapy	1	TEG^®^	TEG^®^: MA > 47 mm	HTPR (MA of 66 mm) at the time of stent thrombosis
Jariwala et al. [78]	Case report	stent thrombosis on ticagleror therapy	1	Not tested	-	stent thrombosis on ticagrelor—stent-related complication excluded by intravascular coronary imaging

ACS—acute coronary syndrome; ADP—adenosine diphosphate; AU—aggregation units; AUC—area under the curve; CAD—coronary artery disease; HTPR—high on treatment platelet reactivity; LTA—light transmission aggregometry; MA—maximum amplitude; NSTE—non-ST segment elevation; PCI—percutaneous coronary intervention; PRI—platelet reactivity index; PRU—P2Y12 reactivity units; T2D—type 2 diabetes; TEG^®^—thromboelastography; VASP-P—Vasodilator-Stimulated Phosphoprotein phosphorylation.

## Data Availability

All the source data are available from the corresponding author upon reasonable request.
